# Response of C57Bl/6 mice to a carbohydrate-free diet

**DOI:** 10.1186/1743-7075-9-69

**Published:** 2012-07-28

**Authors:** Saihan Borghjid, Richard David Feinman

**Affiliations:** 1 Department of Biology, Molloy College, Rockville Centre, NY, 11571, USA; 2 Department of Cell Biology, SUNY Downstate Medical Center, Brooklyn, NY, 11203, USA

## Abstract

High fat feeding in rodents generally leads to obesity and insulin resistance whereas in humans this is only seen if dietary carbohydrate is also high, the result of the anabolic effect of poor regulation of glucose and insulin. A previous study of C57Bl/6 mice (Kennedy AR, et al.: *Am J Physiol Endocrinol Metab* (2007) **262** E1724-1739) appeared to show the kind of beneficial effects of calorie restriction that is seen in humans but that diet was unusually low in protein (5%). In the current study, we tested a zero-carbohydrate diet that had a higher protein content (20%). Mice on the zero-carbohydrate diet, despite similar caloric intake, consistently gained more weight than animals consuming standard chow, attaining a dramatic difference by week 16 (46.1 ± 1.38 g vs. 30.4 ± 1.00 g for the chow group). Consistent with the obese phenotype, experimental mice had fatty livers and hearts as well as large fat deposits in the abdomino-pelvic cavity, and showed impaired glucose clearance after intraperitoneal injection. In sum, the response of mice to a carbohydrate-free diet was greater weight gain and metabolic disruptions in distinction to the response in humans where low carbohydrate diets cause greater weight loss than isocaloric controls. The results suggest that rodent models of obesity may be most valuable in the understanding of how metabolic mechanisms can work in ways different from the effect in humans.

## Introduction

The high fat-fed mouse is a widely investigated model of obesity, insulin-resistance and susceptibility to diabetes. The C57Bl/6 strain, in particular, when subjected to high dietary fat, shows increased consumption, increased efficiency of fat storage (weight gained/calorie) and becomes insulin resistant [1-4]. As a model of human obesity and insulin resistance, however, it suffers from the severe, if under-emphasized, limitation that high-fat diets do not generally cause these conditions in humans unless the diets are also high in carbohydrate. In fact, carbohydrate-restricted diets (CRDs), even with high fat, are the most effective therapy for diabetes and metabolic syndrome; the greater the replacement of carbohydrate with fat, the more effective the improvement (reviews: [5-11]). In humans, it is low-carbohydrate diets that most often demonstrate energy inefficiency [12] with respect to lipogenesis and no diet is better for weight loss although macronutrient effects on energy efficiency remain somewhat controversial.

The rationale for CRDs is that glucose, directly or indirectly through insulin and other hormones, has a catalytic role in controlling other metabolites [10,13]. According to this principle, the observed effect of high fat diets in mice would have to be explained by the presence of sufficient dietary carbohydrate or endogenous insulin to maintain the anabolic state. An influential paper that studied C57BL/6 mice under conditions of zero carbohydrate [14] seemed to bear this out.

Kennedy, *et al.*[14] studied C57Bl/6 mice on a high fat, ketogenic diet (KD) and showed that they displayed all the characteristics of humans on a low-carbohydrate diet: weight loss without caloric restriction, increased energy expenditure, improved glucose tolerance, and lowered serum insulin and leptin levels. The KD in Kennedy, et al.. was 95% energy derived from fat and 5% from protein. Such a diet is rather extreme compared to a diet that might be consumed by a human even in an experimental setting: first, 5% energy from protein is very low and second, whereas there is no dietary requirement for carbohydrate, in humans, the effects attributed to the unique metabolic state associated with carbohydrate restriction have not generally required such an extreme fat/protein ratio. On the other hand, it seemed possible that, because of the small brain size and therefore limited requirements for glucose, very low carbohydrate would be necessary to reproduce in a rodent model the effect of CRDs seen in humans. We have confirmed, in work to be published elsewhere, that moderate lowering of carbohydrate had little effect on mice. We therefore tried to repeat Kennedy’s experiment at more traditional levels of protein while maintaining zero carbohydrate. To our surprise, we observed the phenotype usually associated with the high fat-fed-mouse model. Animals became obese and insulin-resistant and gained substantially more weight per calorie than mice fed normal chow. We describe here the results of these experiments. The outcomes suggest that the results found by Kennedy, *et al.*[14] were a consequence of the low levels of dietary protein, possibly to the point of protein deprivation.

## Material and methods

### Animals

Six-week old male C57Bl/6 mice (The Jackson Laboratory, Maine) were housed individually at 22°C on a 12-hour light–dark cycle. Animals were allowed *ad libitum* access to food, except when fasting overnight as described later. Water was available to the animals at all times. Applicable institutional and governmental regulations concerning the ethical use of animals were followed. All procedures were approved by State University of New York Downstate Medical Center Animal Care and Use Committee.

### Diets and diet trial

Twenty-four six-week-old male C57Bl/6 mice were acclimated to the laboratory conditions while consuming *ad libitum* standard chow (Normal Chow #5001, LabDiet) until they were nine weeks old; at ten weeks, mice are generally considered adult. They were then randomly assigned to two groups and were fed *ad lib* on either 1) a zero-carbohydrate, ketogenic diet (Zero-CHO Group, n = 12), or 2) continuation of the standard chow diet (Chow Group, n = 11). The diet compositions were as follows:

Chow Group: Normal Chow #5001, LabDiet (http://www.labdiet.com/); 3.36 kcal/gm with % energy CHO:fat:protein = 58:13.5:28.5.

Zero-CHO Group: Bio-Serv Inc. (http://www.bio-serv.com/); 6.1 kcal/gm. The macronutrient composition: % energy CHO:fat:protein = 0:80:20. This ketogenic diet was supplemented with vitamin mix and mineral mix that is complete in micronutrients. The fat composition was Lard (401.0 g/kg) and Butter (142.5 g/kg).

The above diets were maintained over a sixteen-week trial period. During the trial period, body weights and *ad libitum* food intake were measured twice weekly. The food intake measurements were corrected for spillage. Physical activity was not monitored but by inspection, as expected, was reduced as animals got fatter.

### Glucose tolerance tests

At the twelfth week of the trial period, all twenty-three mice were fasted overnight and were injected with glucose (2 gram/Kg body weight) intraperitoneally. Tail blood was collected at 0, 15, 30, 60, 120, and 180 min. Blood glucose concentrations were measured using a glucometer (Elite, Bayer).

### Tissue samples

*Retro-orbital Bleeding*: standard heparinized micro-hematocrit capillary tubes were used for blood collection under anesthesia. The blood was withdrawn after overnight fasting, and the amount of blood withdrawn was no more than 1% of the animal’s body weight (e.g., 0.3 ml from a 30gm adult mouse).

*Terminal Bleeding and Necropsy*: after overnight fasting, mice were asphyxiated to unconsciousness using CO_2_ and blood was withdrawn by cardiac puncture. Tissues (brain, heart, liver, skeletal muscle and fat) were collected and snap frozen in liquid nitrogen before storage at −80°C.

Blood serum from retro-orbital bleeding and terminal bleeding was separated by centrifugation at 5000 rpm for 20 min and was stored at −80°C.

### Enzyme-linked immunoSorbent assay

Fasting mouse serum was analyzed using Enzyme-Linked ImmunoSorbent Assay (ELISA). The quantitative determination of serum leptin was done using Mouse Leptin ELISA Kit (Cat #90030, Crystal Chem Inc., IL).

### Statistical analysis

Data are presented as means ± standard error. Statistical significance between groups was assessed by 1) unpaired Student t-Test; 2) one-way ANOVA and Student-Newman-Keuls post hoc analysis; 3) two-way ANOVA. Statistical significance was taken as P < 0.05.

## Results

### Food intake and body weight

**Figure 1:**
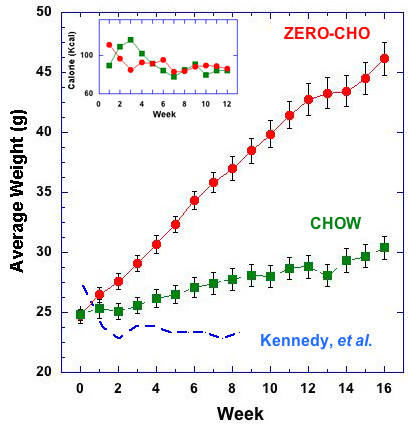
** Average weight as a function of time on the indicated diet.** Body weight and calorie intake (Inset) in male C57Bl/6 mice during 16-week trial period from diet groups: Chow group (green), Zero-CHO group (red). Values are mean ± standard error. The blue dashed line shows body weights of the mice on a ketogenic diet (% energy ratio of CHO:fat:protein = 0:95:5) in the experiment reported by Kennedy that gained less weight than chow-fed mice and whose body weights were similar to calorie restricted (CR) mice that had consumed 65 % of their average chow intake [2]. Statistical differences among the means were determined by one-way ANOVA for calorie intake (P = 0.38) and two-way ANOVA for body weight (P_<_ 0.0001).

In the first week of the trial period, mice introduced to the zero carbohydrate diet (Zero-CHO group; % CHO:fat:protein = 0:80:20) consumed more calories than mice on the standard chow diet (Chow group; % CHO:fat:protein = 58:13.5:28.5). In this period, the Zero-CHO group consumed 105 Kcal compared to 94 Kcal for the Chow group. Over the course of four weeks, differences in the caloric intake of the two groups were minimized and, from week four onward, mice in the Zero-CHO group and Chow group consumed roughly the same total calories weekly (P = 0.38). This pattern of food intake continued throughout the diet trial, which lasted sixteen weeks (Inset to Figure 1).

The mean body weights for each group are shown in Figure 1. Despite the similarity in caloric intake, mice on the Zero-CHO diet consistently gained more weight than mice on standard chow throughout the sixteen-week trial period (P < 0.0001). By week 16, this led to dramatically heavier body weights for mice consuming the Zero-CHO diet (46.1 ± 1.38 g) compared to only 30.4 ± 1.00 g for the Chow group. Consistent with the obese phenotype, mice on Zero-CHO diet had fatty livers and hearts, and large amounts of fat deposition in the abdomino-pelvic cavity (data not shown).

### Impaired glucose clearance

**Figure 2:**
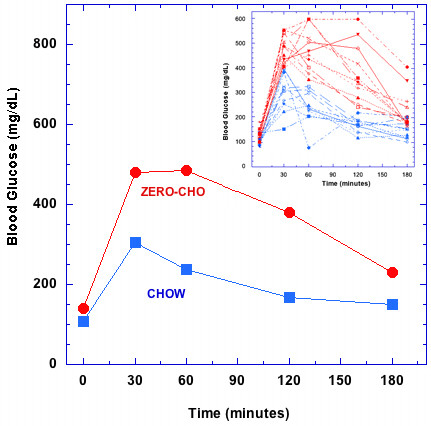
** Glucose tolerance tests on male C57Bl/6 mice fed Zero-CHO and standard Chow diets.** Glucose tolerance tests were administered at week 12 of the diet trial. Blood glucose concentrations are shown at 0 min, 30 min, 60 min, and 180 min time points: Chow group (blue), Zero-CHO group (red). Statistical differences among the means were determined using unpaired Student t-Test at each time point (P<0.0009). The inset to the figure shows that every animal in the zero-CHO group showed decreased glucose clearance.

Glucose tolerance tests revealed impaired glucose clearance in mice fed Zero-CHO diet (Figure 2). Zero-CHO mice had significantly higher fasting glucose levels compared to the Chow fed group at week 12 (Zero-CHO group 138.9 ± 6.62 mg/dL vs. Chow group 107.1 ± 4.30 mg/dL; P < 0.0009). Zero-CHO mice showed significantly higher levels of blood glucose at all time points compared to the Chow group following intraperitoneal glucose injection (P = 0.02). Three hours after glucose injection, Zero-CHO group blood glucose was 91.4 ± 22.95 mg/dL higher than baseline (0 time), more than two-fold increase over the increase of the Chow group (43.8 ± 12.20 mg/dL).

### Leptin

**Figure 3:**
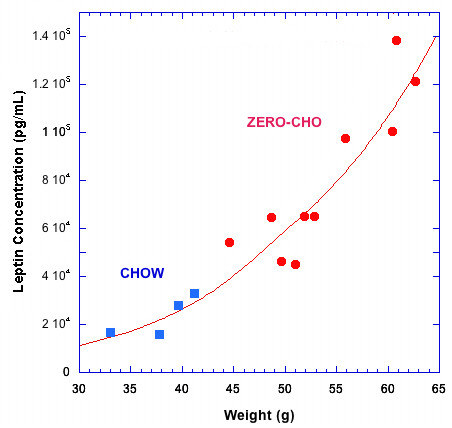
** Correlation of blood leptin level and body weight in male C57Bl/6 mice.** Individual serum leptin concentration of Zero-CHO fed mice (red; n = 10, at 55-week) and Chow fed mice (blue; n = 4, at 57-week). Line represents exponential fit to the plotted points.

Fasting blood leptin levels of male C57Bl/6 mice fed the Zero-CHO and standard Chow diets were measured in a separate study shown in Figure 3. The blood leptin concentration closely correlated with individual mouse’s body weight (R = 0.93). A close correlation between Zero-CHO diet induced obesity and elevation of blood leptin levels is seen in the fact that all of the Zero-CHO mice gained more weight and exhibited higher fasting blood leptin levels than any of the Chow fed mice (Figure 3).

## Discussion

Because of the access to intracellular downstream signaling pathways, animal models provide biological information that is not generally accessible in human. The major caveat in the use of animal models is obviously that different animals have different metabolic responses. This is especially true in diet where humans are omnivores and have substantial flexibility in response to changing macronutrient composition. The “natural” diet of the mouse is dependent on strain but is generally high in carbohydrate and normal is probably operationally defined as laboratory chow. In the context of public health, one must be circumspect in generalizing from the effects in rodents where high-fat diets have an effect that appears to be far less sensitive to carbohydrates than that seen in humans. In particular, low fat recommendations continue to fail in large trials while low carbohydrate diets, even if high in fat, are more effective in isocaloric comparisons for however long they are compared.

Remarkable in the current study is the non-linear caloric response. The mice in this study showed a substantially greater increase in fat per calorie consumed than predicted by the standard “calorie-is-a-calorie” idea frequently invoked in nutrition. This has been reported previously in the literature in C57Bl/6 mice on high-fat diets [15]. This is in distinction to the effect seen in humans where it is frequently found that, on low-carbohydrate diets less fat is gained per calorie, referred to popularly as a “metabolic advantage [16],” that is, the controlling variable is carbohydrate rather than fat. While such non-linear energy efficiency was once claimed to violate laws of thermodynamics, theoretical analysis shows no contradiction from physical law and there are now numerous examples in humans and animal models [12,17]. Animal models of the type studied here make it clear that energy balance is not as assumed. The fact that the inefficiency in humans depends on the level of carbohydrate rather than on fat as seen here in mice emphasizes the need to attend to differences in species.

Differential effect of macronutrients, in this case, saturated *vs.* unsaturated fatty acids was shown by Wen, et al.. [1] who found that the saturated fatty acid palmitate, but not oleate, induced activation of the NLRP3-ASC inflammasome, raising caspase-1, IL-1b and IL-18 production and this impaired insulin signaling in several tissues in culture, reducing glucose tolerance and insulin sensitivity similar to results reported here. However, in humans, Forsythe, *et al.* showed that compared to the response to a low fat diet (LFD) (% CHO:fat:protein = 56:24:20) a low-carbohydrate, high-fat diet (% CHO:fat:protein = 12:59:28) with three times the amount of saturated fat as the LFD led to an increased proportion of serum total n-6 PUFA, mainly attributed to a marked increase in arachidonate (20:4n-6). The n-6/n-3 and arachidonic/eicosapentaenoic acid ratio also increased sharply. Both diets significantly decreased the concentration of several serum inflammatory markers, but there was an overall greater anti-inflammatory effect associated with the very low carbohydrate ketogenic diet, as evidenced by greater decreases in TNF-alpha, and numerous other markers. Increased 20:4n-6 and the ratios of 20:4n-6/20:5n-3 and n-6/n-3 are commonly viewed as pro-inflammatory but, unexpectedly, were consistently inversely associated with responses in inflammatory proteins [18,19].

## Conclusions

At least in the widely used C57Bl/6 mouse, high-fat feeding, even in the absence of dietary carbohydrate, leads to obesity and an associated increase in leptin levels as well as distinct insulin resistance. This is in distinction to the results of Kennedy, *et al.*[14] whose ketogenic diet in C57Bl/6 mice more closely resembled the beneficial state found in humans on carbohydrate-restricted diets. The likely explanation is that that diet was protein-deficient and the mice were not in a normal metabolic state. In the end, the great power of modern molecular biology in rodent models may provide more evidence as to the mechanisms by which humans are different from rodents in the response to diet.

## **Competing interest**

The authors declare that they have no competing interests.

## **Authors’ contribution**

SB designed and carried out the experimental protocol. Both authors conceived the experiment, analyzed the data and wrote the manuscript. Both authors read and approved the final manuscript.
